# Mechanisms of signal transduction by ethylene: overlapping and non-overlapping signalling roles in a receptor family

**DOI:** 10.1093/aobpla/plt010

**Published:** 2013-03-28

**Authors:** Samina N. Shakeel, Xiaomin Wang, Brad M. Binder, G. Eric Schaller

**Affiliations:** 1 Department of Biological Sciences, Dartmouth College, Hanover, NH 03755, USA; 2 Department of Biochemistry, Quaid-i-azam University, Islamabad 45320, Pakistan; 3 Department of Biochemistry and Cellular & Molecular Biology, University of Tennessee, Knoxville, TN 37996, USA

**Keywords:** *Arabidopsis*, ethylene, ethylene receptors, histidine kinase, hormone signalling, sub-functionalization, two-component system

## Abstract

The plant hormone ethylene regulates growth and development as well as responses to biotic and abiotic stresses. Over the last few decades, key elements involved in ethylene signal transduction have been identified through genetic approaches, these elements defining a pathway that extends from initial ethylene perception at the endoplasmic reticulum to changes in transcriptional regulation within the nucleus. Here, we present our current understanding of ethylene signal transduction, focusing on recent developments that support a model with overlapping and non-overlapping roles for members of the ethylene receptor family. We consider the evidence supporting this model for sub-functionalization within the receptor family, and then discuss mechanisms by which such a sub-functionalization may occur. To this end, we consider the importance of receptor interactions in modulating their signal output and how such interactions vary in the receptor family. In addition, we consider evidence indicating that ethylene signal output by the receptors involves both phosphorylation-dependent and phosphorylation-independent mechanisms. We conclude with a current model for signalling by the ethylene receptors placed within the overall context of ethylene signal transduction.

## Abstract (precis)

The plant hormone ethylene regulates growth and development as well as stress responses. This review focuses on recent discoveries that support a model for ethylene signal transduction that involves overlapping and non-overlapping roles for members of the ethylene receptor family. The roles of ethylene receptors in regulating plant growth, pathogen responses, and development are discussed. Mechanisms are proposed by which receptors can modulate downstream responses together and independently.

## Introduction and Background

The gaseous hormone ethylene plays multiple roles in regulating plant growth and development (reviewed in Abeles *et al.* 1992). In terms of growth, ethylene is most commonly associated with the regulation of cell size, particularly as an inhibitor of cell elongation. However, ethylene may also serve as a signal to promote cell expansion, an important response to submergence stress in some species (reviewed in Jackson 2008). In addition to regulating cell expansion, ethylene has also been found to regulate growth through control of cell division in some instances. In terms of development, ethylene is most commonly associated with ‘ageing’, particularly for its ability to accelerate such processes as senescence, ripening and abscission (reviewed in Stepanova and Alonso 2009; Schaller 2012). In addition, ethylene serves as a key modulator of the plant's response to biotic or abiotic stresses.

**Figure 1.**
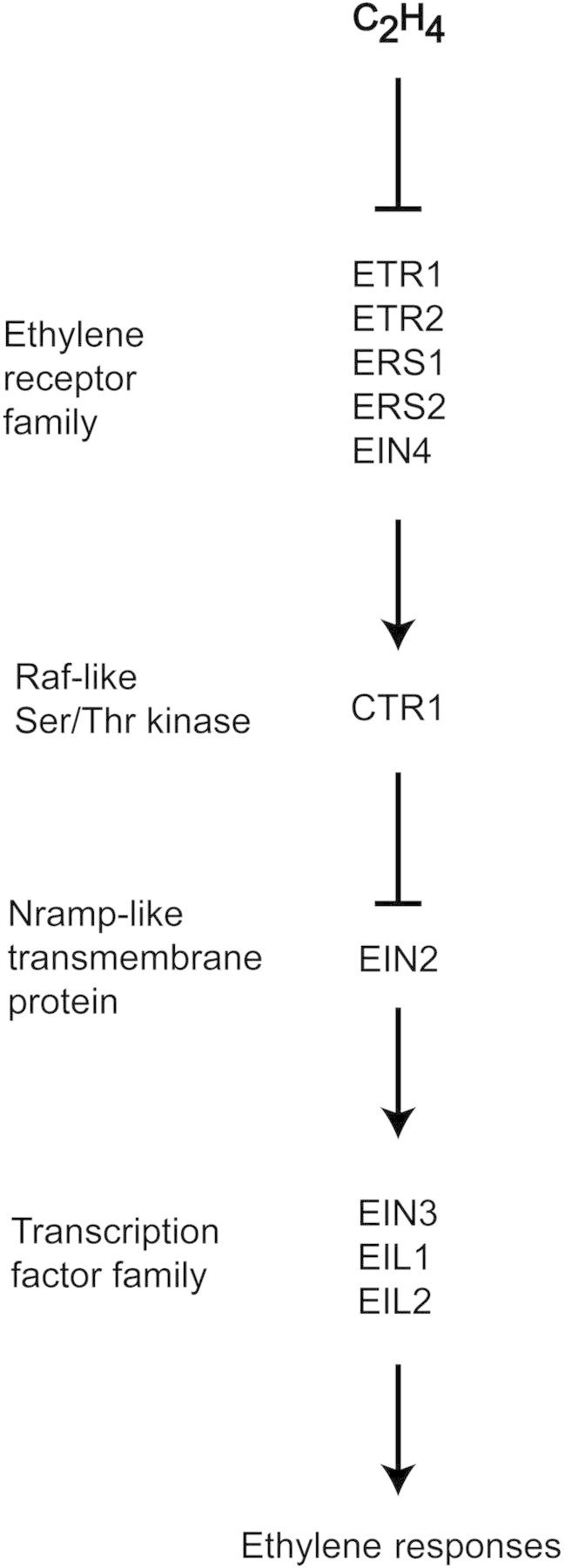
Genetically defined pathway for ethylene signal transduction. Genes in the primary signal transduction pathway are shown. The order of action is based on double-mutant analysis.

Genetic analysis conducted over the past couple of decades, primarily with the model plant *Arabidopsis*, has resulted in the identification of key elements that mediate the primary response to ethylene (reviewed in Klee 2004; Chen *et al.* 2005). Characterization of these signalling elements has resulted in a model for ethylene signal transduction that is essentially a linear pathway, initiated by ethylene binding to membrane-bound receptors and culminating in transcriptional regulation at the nucleus (Fig. 1). Interestingly, the ethylene receptors as well as the initial signalling elements in the pathway are predominantly localized to the endoplasmic reticulum (ER) (reviewed in Ju and Chang 2012). The ER is an unusual location for a hormone receptor but is compatible with the ready diffusion of ethylene in aqueous and lipid environments.

In *Arabidopsis*, ethylene is perceived by a five-member family of ethylene receptors, the founding member being the ethylene receptor ETR1 (Fig. 1). Genetic studies demonstrate that the receptors act as negative regulators in the ethylene-signalling pathway (Hua and Meyerowitz 1998; Wang *et al.* 2003; Qu and Schaller 2004). In the absence of ethylene, the receptors activate CTR1, a Ser/Thr kinase that suppresses the ethylene response (Kieber *et al.* 1993; Clark *et al.* 1998). The direct phosphorylation target of CTR1 is EIN2, an ER-bound protein with similarity to Nramp metal-ion transporters (Alonso *et al.* 1999), which is maintained in an inactive state when phosphorylated by CTR1 (Ju *et al.* 2012; Qiao *et al.* 2012). Upon ethylene binding, the receptors inactivate CTR1, thereby relieving the suppression on the downstream signalling elements. As a result, EIN2 is proteolytically processed such that its C-terminal domain is released to migrate to the nucleus (Ju *et al.* 2012; Qiao *et al.* 2012; Wen *et al.* 2012). In the nucleus, EIN2 either directly or indirectly activates the transcription factors EIN3 and EIN3 like1 (EIL1) to initiate the transcriptional response to ethylene (Chao *et al.* 1997; Solano *et al.* 1998; Alonso *et al.* 2003).

**Figure 2.**
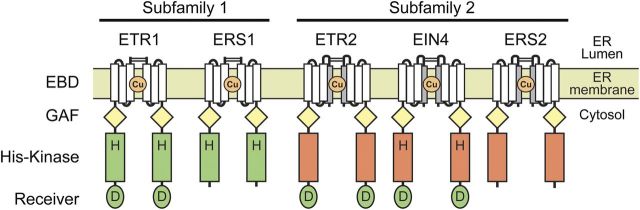
Ethylene receptor family of *Arabidopsis.* The ethylene receptor family of *Arabidopsis* is divided into subfamilies 1 and 2 based on phylogenetic analysis and structural features. Receptors are shown as homo-dimers. The ethylene-binding domain (EBD) is found within the conserved transmembrane domains (white rectangles), and includes a copper cofactor (Cu); subfamily 2 receptors have an additional predicted transmembrane domain (grey rectangle) that may function as a signal sequence. All five members of the ethylene receptor family have a GAF domain (yellow diamond) implicated in protein–protein interactions. His kinase domains are indicated by green or red rectangles, green indicating a functional His kinase domain and red indicating a diverged His kinase domain. The receiver domains (ovals) have the conserved residues required for function and are therefore coloured green. Conserved His (H) and Asp (D) phosphorylation sites are indicated if present.

This whole process of signal transduction is initiated by the binding of ethylene to its receptors. In all plants examined to date, including monocots, dicots and the moss *Physcomitrella patens*, the ethylene receptors exist as a multi-member family (reviewed in Binder *et al.* 2012). The *Arabidopsis* ethylene receptor family is composed of five members, ETR1, ERS1, ETR2, ERS2 and EIN4, all of which have a similar modular structure (Fig. 2). They contain three conserved transmembrane domains near the N-terminus, the transmembrane region also encompassing the ethylene-binding site (Schaller and Bleecker 1995; Rodriguez *et al.* 1999; O'Malley *et al.* 2005). These are followed by a GAF domain, which may mediate protein–protein interactions (Xie *et al.* 2006; Gao *et al.* 2008; Grefen *et al.* 2008). Then, in the C-terminal portion of the receptors, there are domains related to histidine (His) kinases and receiver domains, the so-called ‘two-component’ signalling elements common to prokaryotic signal transduction (reviewed in Schaller *et al.* 2008,^2011^).

The ethylene receptors can be divided into two subfamilies based on phylogenetic analysis and some shared structural features (reviewed in Bleecker 1999; Chang and Stadler 2001; Schaller and Kieber 2002). In *Arabidopsis*, subfamily 1 is comprised of ETR1 and ERS1, and subfamily 2 is comprised of ETR2, ERS2 and EIN4. The primary characteristic that distinguishes the two subfamilies is that subfamily 1 receptors each have a conserved His kinase domain, while subfamily 2 receptors each have a diverged His kinase domain that lacks residues essential for this activity. In addition, the subfamily 2 receptors contain an extra transmembrane domain at the N-terminus, which may function as a signal sequence.

The coupling of biochemical, cellular and genetic approaches has facilitated the development of a more mechanistic model for how the ethylene receptors function. The receptors are predominantly localized to the ER, with a topology that places the N-terminus within the ER lumen, the N-terminal transmembrane domains within the ER membrane itself and the large C-terminal soluble domains within the cytosol (Chen *et al.* 2002, 2007, 2010; Ma *et al.* 2006; Dong *et al.* 2008; Grefen *et al.* 2008; Zhong *et al.* 2008). The ethylene receptors exist as homodimers, the dimeric form stabilized by disulfide bonds between Cys residues found at the N-terminus (Schaller and Bleecker 1995; Hall *et al.* 2000; Gao *et al.* 2008; Chen *et al.* 2010). Interestingly, given the presence of five receptor isoforms in *Arabidopsis*, the homodimeric forms appear to predominate *in planta*, although higher-order associations among the homodimers can occur, leading to heteromeric clusters of the receptors (Gao *et al.* 2008). The dimeric form of the receptor is apparently critical to function because (i) there appears to be a single ethylene-binding site per dimer and (ii) the evolutionarily related His kinases of prokaryotes require a dimeric form for signal output (Schaller *et al.* 1995; Rodriguez *et al.* 1999). The ethylene-binding site contains a copper cofactor, which is critical for ethylene binding (Schaller and Bleecker 1995; Rodriguez *et al.* 1999), and which is made available to the receptors through action of the copper transporter RAN1 (Hirayama *et al.* 1999; Woeste and Kieber 2000; Binder *et al.* 2010).

The above description of ethylene signal transduction suggests a fairly simple linear pathway. But such a description is difficult to reconcile with the ability of plants to respond to ethylene across a concentration range spanning over six orders of magnitude, as well as the role of ethylene in regulating such a wide variety of downstream responses (Chen and Bleecker 1995; Binder *et al.* 2004). In this report, we discuss recent progress in our understanding of signalling by ethylene receptors that provides at least a partial answer to these problems, proposing in effect that the pathway is not as linear as initial genetic studies suggested. To this end, we discuss the evidence supporting overlapping and non-overlapping roles for the receptors in signalling. We then consider mechanisms by which such sub-functionalization may occur, discussing (i) the importance of receptor interactions in modulating signal output and (ii) the significance of enzymatic activity in signal output by the receptors. We conclude with a current model for signalling by the ethylene receptors placed within the overall context of ethylene signal transduction.

## Overlapping and Non-overlapping Roles of the Ethylene Receptors

Ethylene receptors exist as multi-member families in plants, which can be divided into two subfamilies (Fig. 2). However, there are differences among the individual members of the subfamilies that exist even beyond a simple evolutionary divergence in amino acid sequence. For instance, in subfamily 1 of *Arabidopsis*, the receptor ETR1 contains a receiver domain but ERS1 does not (Fig. 2). Similarly, the receptors ETR2 and EIN4 of subfamily 2 have receiver domains but ERS2 does not. In addition, even though the subfamily 2 receptors all have diverged His kinase domains, where these divergences occur varies among the receptors. In particular, EIN4 has retained the conserved His for phosphorylation, whereas the other subfamily 2 members have not. The sequence and domain differences among the receptors are suggestive of functional differences. Here we discuss recent data indicating that, in addition to their general role in ethylene perception, the receptors may also play more individualistic roles in transducing the ethylene signal.

### Overlapping roles of the receptors in the regulation of ethylene growth responses

The initial identification and characterization of the *Arabidopsis* ethylene receptors was based, not surprisingly, on their ability to modulate a common set of well-characterized ethylene responses, in particular the ‘triple response’ of dark-grown seedlings to ethylene (Bleecker *et al.* 1988; Guzman and Ecker 1990; Chang *et al.* 1993; Hua *et al.* 1995, 1998; Roman *et al.* 1995; Sakai *et al.* 1998). The *Arabidopsis* triple response is characterized by (i) an inhibition of hypocotyl and root elongation, (ii) an increase in radial swelling of the hypocotyl, and (iii) the formation of an exaggerated apical hook (Bleecker *et al.* 1988; Guzman and Ecker 1990). By exploiting the triple response in a genetic screen, the ethylene-insensitive *etr1-1* mutant was isolated, which led to the identification of the receptor ETR1 (Bleecker *et al.* 1988; Chang *et al.* 1993; Schaller *et al.* 1995). Dominant ethylene-insensitive mutations in the receptors ETR2, ERS1, ERS2 and EIN4 further confirmed an overlapping role in control of the various growth effects described as the triple response (Hua *et al.* 1995; Hua and Meyerowitz 1998; Sakai *et al.* 1998).

**Figure 3.**
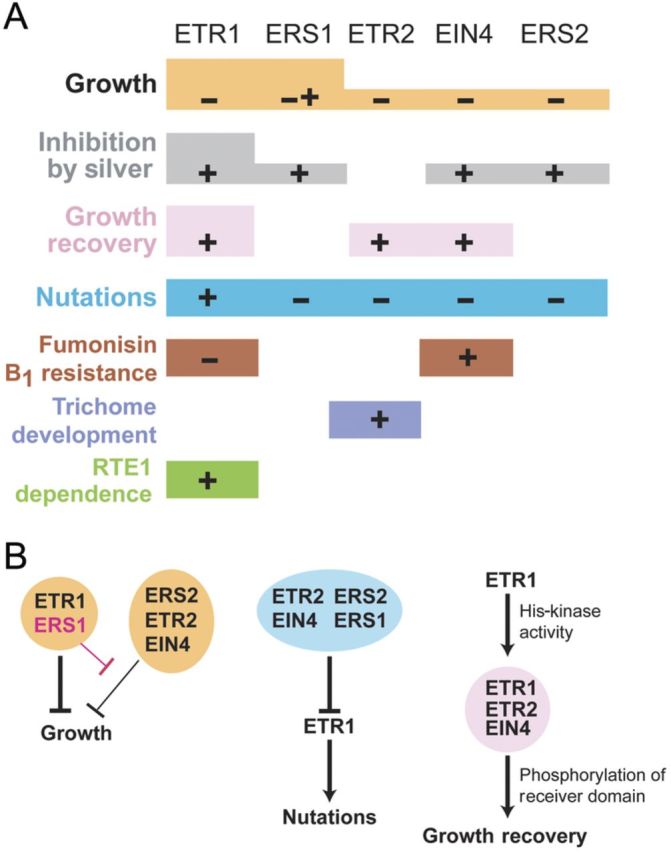
Overlapping and non-overlapping functions within the ethylene receptor family. (A) Roles of individual receptors in ethylene-regulated responses. A plus sign (+) indicates that the receptor activates the response, a negative sign (−) that the receptor inhibits the response and both signs (− +) that the receptor has differing effects dependent on other factors. The lack of a sign indicates that the receptor has no observed effect on the response. Differences in the box height indicate differences in receptor contribution to a particular response. (B) Details of interactions among receptors for selected ethylene responses. Differing thickness of arrows indicates differing contributions to a response.

The isolation and characterization of loss-of-function (LOF) mutations in the ethylene receptors made it clear that there was a substantial level of functional overlap among members of the ethylene receptor family (Hua and Meyerowitz 1998). Single and some double LOF mutants exhibited an ethylene response similar to that of wild-type seedlings. However, higher-order mutants lacking multiple members of the ethylene receptor family exhibited a constitutive ethylene-response phenotype when grown in the air (i.e. without ethylene). The constitutive ethylene-response phenotype was observed when seedlings were grown in either the dark or the light. This study thus demonstrated that the receptors (i) were negative regulators of the ethylene response and (ii) had overlapping function in the control of ethylene responses. A limitation of this pioneering study by Hua and Meyerowitz (1998) was that LOF mutant combinations for only four of the five receptors could be analysed, no LOF mutation in the subfamily 1 receptor ERS1 then being available. This situation was subsequently remedied with the isolation of partial LOF (Zhao *et al.* 2002; Hall and Bleecker 2003; Wang *et al.* 2003) and null (Qu *et al.* 2007) mutations of *ERS1*. Characterization of the *etr1 ers1* double mutants demonstrated that they had a constitutive ethylene-response phenotype stronger than any mutant combination previously characterized. These data were consistent with overlapping function among the ethylene receptors but also indicated that, in *Arabidopsis*, the subfamily 1 receptors play a more predominant role than the subfamily 2 receptors in mediating the well-characterized ethylene growth responses (Fig. 3). Of particular significance was the finding that the constitutive ethylene-response phenotype of an *etr1 ers1* double mutant could be rescued by subfamily 1 receptors but not by subfamily 2 receptors, clearly establishing a functional difference between the two receptor subfamilies (Wang *et al.* 2003).

Although genetic studies generally support the receptors functioning as negative regulators of ethylene signal transduction, recent studies with mutations in *ERS1* suggest that more complex interactions among the receptors can also occur (Liu *et al.* 2010). In particular, it was found that the addition of an *ers1* LOF mutation to any receptor mutant background that contained wild-type *ETR1* partially reversed the mutant phenotype (Fig. 3). For example, the subfamily 2 triple mutant *etr2 ein4 ers2* has a stronger constitutive ethylene-response phenotype than does the quadruple *ers1 etr2 ein4 ers2* mutant. These results demonstrate that *ERS1* can act as either a negative or a positive regulator of the ethylene response depending on the genetic background.

### Sub-functionalization of the receptors

#### ETR1 plays a dominant role in mediating the effects of silver on ethylene perception

The availability of LOF and gain-of-function (GOF) mutations for all five ethylene receptors of *Arabidopsis* has facilitated their functional characterization in an ever-expanding array of ethylene-mediated responses. These analyses support sub-functionalization within the receptor family such that subsets or individual receptors play a predominant role in mediating specific effects of ethylene (Fig. 3). For example, there are non-overlapping roles for the receptors in mediating the inhibitory effects of silver on ethylene perception. Silver ions inhibit ethylene responses in plants, yet support ethylene-binding activity in ETR1 (Beyer 1976). This led to the hypothesis that Ag(I) occupies the ethylene/metal-binding pocket of ETR1, but upon binding of ethylene does not allow for signal transmission through the receptors (Rodriguez *et al.* 1999; Binder *et al.* 2007). However, it was recently shown that ETR1 is sufficient and plays a dominant role in mediating the effects of silver ions (McDaniel and Binder 2012). Most notably, *etr1* LOF mutants had reduced responses to AgNO_3_; in other words, they have partial responses to ethylene in the presence of AgNO_3_. By contrast, other receptor LOF mutants had unaltered responses to AgNO_3_ so that ethylene failed to inhibit growth in these plants when AgNO_3_ was present. The result with *etr1* LOF mutants was not simply a case of higher ETR1 levels leading to a predominant role, since the mRNA abundances of ETR1 and ERS1 are similar in dark-grown seedlings (Binder *et al.* 2004). Thus, ETR1 has a unique function in this trait. Additionally, AgNO_3_ supported ethylene-binding activity in ETR1 and ERS1, but not the subfamily 2 receptors. However, complementation studies showed that all the receptor isoforms, except ETR2, can rescue the silver phenotype, providing additional evidence for sub-functionalization of the receptors (McDaniel and Binder 2012).

#### A subset of receptors regulates growth recovery following exposure to ethylene

Differences have been found in the ability of receptors to mediate growth recovery following exposure to ethylene (Fig. 3). Using time-lapse imaging of growing seedlings it was found that LOF mutants lacking any of the receptors with a receiver domain (ETR1, ETR2, EIN4) had slow growth recovery after the removal of ethylene (Kim *et al.* 2011). The triple *etr1 etr2 ein4* LOF mutants had severely delayed growth recovery, whereas *ers1 ers2* double LOF mutants had unaltered growth recovery (Kim *et al.* 2011).

#### Differing receptor roles in nutational bending

Differential growth leading to nutational bending has also been linked to ethylene. Nutations in the apical hook are oscillatory movements resulting from localized and differential growth (Berg and Peacock 1992). Mutational analysis showed that ETR1 was both necessary and sufficient for ethylene-stimulated nutational bending of the apical hook since eliminating ETR1 (either singly or in combination) eliminated ethylene-stimulated nutations (Binder *et al.* 2006). By contrast, removing all four of the other receptor isoforms led to constitutive nutations (Kim *et al.* 2011), showing that ETR1 has the opposite role to the other isoforms in controlling this trait (Fig. 3). Ethylene-stimulated nutations do not require ETR1 His kinase activity but do require a full-length ETR1 protein, suggesting that protein–protein interactions could be important in this trait (Kim *et al.* 2011).

#### An individualized role of receptors in pathogen responses

Ethylene production is often stimulated by pathogen attack and is an important part of the plant's response to pathogen attack (Abeles *et al.* 1992). However, the role of ethylene in responses to pathogens is complex and depends upon the pathogen involved. For instance, tobacco plants transformed with the *etr1-1* transgene were ethylene insensitive, leading to plants that were susceptible to normally non-pathogenic fungi, but had normal responses to tobacco mosaic virus infection (Knoester 1998). This complexity suggests that ethylene is likely to have multiple roles in pathogen responses. Fumonisin B_1_ is a fungal toxin that induces cell death. One study using ethylene GOF mutants showed that the *etr1-1 Arabidopsis* mutants were more susceptible to fumonisin B_1_ than wild-type plants; by contrast, *ein4-1* mutants were less susceptible to fumonisin B_1_ (Plett *et al.* 2009*a*). The other receptor isoform mutants had responses to the toxin similar to those of wild-type plants, showing that ETR1 and EIN4 have isoform-specific, and opposite, roles in mediating responses to fumonisin B_1_. These results are intriguing because all of the receptor mutants used in this study were ethylene insensitive (Hua and Meyerowitz 1998; Hua *et al.* 1998; Sakai *et al.* 1998). This suggests that there is unique signalling from ETR1 that needs to be turned off by ethylene to reduce susceptibility to fumonisin B_1_ toxin, whereas there is unique signalling from EIN4 that decreases susceptibility to the toxin and plants are more susceptible when EIN4 is turned off in response to ethylene. It is currently unknown whether these unique roles are due to differences in enzymatic output or receptor–protein interactions, or both.

#### A role for ETR2 in trichome development

In an examination of ethylene receptor GOF and LOF mutants, only *etr2* mutants had altered trichome and root hair branching (Plett *et al.* 2009*b*). The *etr2* GOF mutants had wild-type trichome branching, but the *etr2* LOF mutants had a higher number of trichomes containing two branches. This trait was not limited to trichomes since the roots of the *etr2* GOF mutants had fewer branched root hairs than wild-type plants; in contrast, *etr2* LOF mutants had a higher percentage of branched root hairs (Plett *et al.* 2009*a*, *b*). Interestingly, the trichomes of *etr2* LOF mutants had altered expression patterns for MICROTUBULE-ASSOCIATED PROTEIN 4, providing evidence that of the five receptor isoforms, ETR2 uniquely affects microtubule assembly (Plett *et al.* 2009*b*).

### What mechanisms underlie sub-functionalization of the ethylene receptors?

Together, the above studies support a growing body of evidence that the ethylene receptors have both overlapping and non-overlapping roles in *Arabidopsis*. This is not unique to *Arabidopsis* since specific receptor isoforms have a predominant role in fruit ripening in tomato (Tieman *et al.* 2000; Kevany *et al.* 2007), responses to salt stress in tobacco (Chen *et al.* 2009) and the growth and development of rice (Wuriyanghan *et al.* 2009). In only a few cases has the mechanistic basis for these differences been uncovered, but differences in signal output domains of the receptors are likely to be critical to their sub-functionalization. More specifically, sequence differences can result in differing affinities for associated signalling factors or even an association with isoform-specific factors, a possibility we explore in the following section on receptor interactions. Furthermore, the differences in enzymatic activity of subfamily 1 and subfamily 2 receptors potentially allow for regulation of different downstream factors, a possibility we explore in the section thereafter on output from the receptors.

## The Importance of Receptor Interactions in the Regulation of Ethylene Signal Transduction

The initial players mediating the primary response to ethylene—the ethylene receptors, CTR1 and EIN2—all localize to the ER (Chen *et al.* 2002, 2007; Gao *et al.* 2003; Grefen *et al.* 2008; Bisson *et al.* 2009). Perhaps not surprisingly, evidence is accruing that all these players physically interact with each other to form a large multimeric signalling complex at the ER (reviewed in Ju and Chang 2012). The minimal functional unit for the ethylene receptors is a disulfide-linked dimer, primarily a homo-dimer based on *in planta* analysis (Schaller *et al.* 1995; Hall *et al.* 2000; Gao *et al.* 2008), but these receptor homo-dimers form higher-order complexes with each other, the receptor clusters potentially allowing for cross-talk and signal amplification, as has been found with the His kinase-linked chemoreceptors of prokaryotes (Gao *et al.* 2008; Grefen *et al.* 2008). The receptors also physically interact with CTR1, this interaction being required for the localization of CTR1, a protein with no transmembrane domains, to the ER (Gao *et al.* 2008; Zhong *et al.* 2008). Finally, although not as well characterized as the above interactions, recent data also indicate that the receptors associate with EIN2 (Bisson *et al.* 2009; Bisson and Groth 2010). The general significance of these interactions has recently been reviewed (Ju and Chang 2012). Here we consider the importance of receptor interactions in modulating their signal output and how such interactions vary in the receptor family. To this end, we consider (i) the differing interactions of the receptors with CTR1, (ii) the specific interaction of the receptor ETR1 with the regulatory protein RTE1, and (iii) evidence that as yet undiscovered proteins may account for some of the non-overlapping functions among the ethylene receptor family.

CTR1 functions as a key mediator of ethylene signal transduction, acting just downstream of the receptors in transmitting the ethylene signal (Kieber *et al.* 1993). Evidence indicates that all five ethylene receptors from *Arabidopsis* physically interact with CTR1 (Clark *et al.* 1998; Cancel and Larsen 2002; Gao *et al.* 2003) but this does not necessarily imply that all interactions are equivalent. For instance, yeast two-hybrid analysis indicates that the strength of interactions between the ethylene receptors and CTR1 varies depending on several factors. First, CTR1 interacts more strongly with the subfamily 1 receptors ETR1 and ERS1 than it does with the subfamily 2 receptor ETR2 (Clark *et al.* 1998; Cancel and Larsen 2002). Second, interaction of CTR1 with the receptors involves not just the receptor kinase domain but also the receptor receiver domain (Clark *et al.* 1998). Since only a subset of the receptors have the receiver domain, this interaction represents a mechanism by which CTR1 may be regulated differentially within the receptor family.

Several studies also indicate that the amount of CTR1 recruited to the ER by the receptors does not always correlate with the amount of signal transmission from the receptor/CTR1 complexes. Null mutations in *ETR1*, surprisingly, result in an increase in the level of membrane-associated CTR1, the opposite of what one would predict given the receptor dependence for membrane association (Gao *et al.* 2003; Qu *et al.* 2007). If all receptors activate CTR1 equivalently, such an increase in CTR1 levels is predicted to result in a stronger suppression of the ethylene response. But, conflicting with this model, one finds that seedlings null for *ETR1* actually exhibit increased ethylene sensitivity (Cancel and Larsen 2002; Qu *et al.* 2007). In a more recent study, it was found that kinase-inactive versions of ETR1 recruited less CTR1 to the membrane than wild-type versions of ETR1 (Hall *et al.* 2012). Again, if the level of CTR1 correlates with the level of signal output from the receptors, the prediction is that the kinase-inactive lines would exhibit increased ethylene sensitivity. But, again conflicting with such a model, the plants with low levels of CTR1 actually suppress ethylene responses more strongly than those with the higher level of CTR1 (Hall *et al.* 2012). These studies point to ETR1 activating CTR1 more effectively than other members of the receptor family, in particular more effectively than the subfamily 2 receptors, and also suggest that the kinase activity of ETR1 may play a role in this activation, a point we will return to later in this review. An ability of the subfamily 1 receptors to activate CTR1 more effectively than the subfamily 2 receptors may explain the predominant role of the subfamily 1 receptors in regulation of ethylene signalling in *Arabidopsis* (Wang *et al.* 2003; Qu *et al.* 2007).

The interaction of the ethylene receptor ETR1 with the regulatory protein RTE1 is the best example of how a protein interaction can play an isoform-specific role in ethylene signalling (Fig. 3). *RTE1* was identified through a genetic screen as a gene required for the dominant ethylene insensitivity conferred by the GOF *etr1-2* mutation (Resnick *et al.* 2006). *RTE1* encodes a transmembrane protein that physically associates with ETR1 (Dong *et al.* 2008, 2010). Several lines of genetic evidence support the hypothesis that the effects of RTE1 occur predominantly due to an isoform-specific interaction with ETR1. First, an *rte1* LOF mutant phenocopies an *etr1* LOF mutant, both mutants exhibiting an enhanced sensitivity to ethylene (Resnick *et al.* 2006; Zhou *et al.* 2007). Second, overexpression of *RTE1* results in reduced ethylene sensitivity and, significantly, this overexpression phenotype is largely dependent on the presence of *ETR1* (Resnick *et al.* 2006; Zhou *et al.* 2007). Third, the mutations such as *etr1-2*, where RTE1 is important for conferring dominant ethylene insensitivity, are specific to ETR1 and do not lead to ethylene insensitivity when introduced into other members of the ethylene receptor family (Resnick *et al.* 2006; Rivarola *et al.* 2009). The role of RTE1 appears to be to stabilize or assist folding of ETR1 into the conformation it adopts in air (absence of ethylene), thereby enhancing the ability of ETR1 to repress ethylene signalling. Interestingly, the *RTE1* transcript is up-regulated by ethylene (Resnick *et al.* 2006), suggesting that *RTE1* may facilitate the adaptation response of plants to ethylene. For example, a low level of ethylene would induce the production of RTE1, which through its interactions with ETR1 would desensitize the receptor to ethylene, essentially re-setting the plant to now respond to a higher ethylene concentration. A specific regulator for ETR1 activity may have arisen due to the substantial role that ETR1 plays in mediating the *Arabidopsis* ethylene response.

Recent evidence suggests that RTE1 and ETR1 may function together to mediate ethylene signalling through a CTR1-independent pathway. Qiu *et al*. (2012) found that expression of the N-terminal half of ETR1 could partially suppress the constitutive ethylene-response phenotype of a *ctr1* mutant. This intriguing result supports the existence of alternative pathway(s) to the well-known one involving CTR1, and furthermore indicates that the N-terminal half of ETR1 can mediate signal output through such an alternative CTR1-independent pathway. This effect of the N-terminal half of ETR1 was dependent on the presence of RTE1, emphasizing its importance in mediating an isoform-specific output from ETR1 (Qiu *et al.* 2012). Here, the role of RTE1 may simply be to maintain a particular conformation of ETR1 required for input into this alternative pathway. The possibility exists, however, that ETR1 signals to this alternative pathway through RTE1 itself. Most topological prediction programs (e.g. TopPred and TMpred) predict transmembrane domains near the N- and C-termini of RTE1, placing the central soluble portion of RTE1 within the ER lumen (Hofmann and Stoffel 1993; Claros and von Heijne 1994). Such a topology would potentially allow RTE1 to regulate events occurring within the ER, and raises the possibility that ETR1 could have signal outputs to both the cytosol and the ER lumen.

Other plants contain genes similar to RTE1, the best characterized of these being the *GREEN-RIPE (GR)* gene of tomato. *GREEN-RIPE* was identified due to a mutation that resulted in its overexpression and consequent inhibition of tomato ripening (Barry and Giovannoni 2006). Both tomato and *Arabidopsis* contain additional *RTE1*/*GR*-like genes, and a recent comparative analysis of *GR* with its closest tomato homologue, *GRL1*, suggests that sub-functionalization has occurred within this gene family (Ma *et al.* 2012). *GREEN-RIPE* and *GRL1* differ in their abilities to inhibit subsets of the ethylene response when overexpressed in tomato: *GR* but not *GRL1* inhibiting fruit ripening, *GRL1* but not *GR* affecting the petiole epinasty response to ethylene. Over-expressed *GR* and *GRL1* exhibit similar inhibitory effects on the root and hypocotyl growth response to ethylene as well as ethylene-induced petal abscission, these overlapping functions being accentuated when both genes are overexpressed together. These overlapping and non-overlapping functions for GR and GRL1 could potentially arise due to isoform-specific interactions with the tomato ethylene receptor family (Ma *et al.* 2012).

Other isoform-specific mediators of ethylene responses, besides RTE1, are likely to exist in plants. Evidence for this hypothesis comes from gel filtration analysis of ethylene receptor complexes solubilized from *Arabidopsis* (Chen *et al.* 2010). Based on this analysis, there is a substantial degree of heterogeneity among the ethylene receptor complexes, with different members of the receptor family forming complexes of different sizes. The size of the ERS1 protein complex changed dynamically in response to ethylene treatment, indicating that some proteins reversibly associate with the receptor upon ligand binding; this ligand-dependent change in the receptor complex was not observed with the other subfamily 1 receptor ETR1. Evidence from this study indicates that many of these proteins associated with the receptor complexes are likely to represent novel components, the heterogeneity among the complexes suggesting that these as yet unidentified components could play a role in tailoring individual receptors to particular cellular tasks.

## Signal Output by the Receptors

As with many signal transduction systems, protein kinases play a key role in mediating ethylene signalling. What is unusual in the ethylene-signalling pathway is that two types of kinase of disparate evolutionary origin function in concert. The ethylene receptors are related to the His kinases prevalent in prokaryotic signalling systems (reviewed in Chang and Meyerowitz 1995; Schaller *et al.* 2008), whereas CTR1 is a Ser/Thr kinase most closely related to the mitogen-activated protein kinase kinase kinases (MAPKKKs) of eukaryotic systems (Kieber *et al.* 1993; Huang *et al.* 2003). The ethylene receptors and CTR1 physically interact, forming a protein complex that mediates the initial response to ethylene binding (Clark *et al.* 1998; Cancel and Larsen 2002; Gao *et al.* 2003; Zhong *et al.* 2008). Mutations in the kinase domain of CTR1 demonstrate that its enzymatic activity is essential to function (Kieber *et al.* 1993; Huang *et al.* 2003) and, indeed, one of the substrates for CTR1 is EIN2, the next downstream element in the signalling pathway (Ju *et al.* 2012). On the other hand, in spite of the homology of the ethylene receptors to His kinases, this enzymatic activity is largely dispensable for many of the well-characterized ethylene responses (Wang *et al.* 2003; Hall *et al.* 2012). Instead, transmission of the ethylene signal from receptors to CTR1 appears to occur predominantly by a phosphorylation-independent mechanism, potentially by propagation of conformational changes within the ethylene receptor/CTR1 complex (reviewed in Binder *et al.* 2012). Here we discuss data on how kinase activity of the receptors, while not being required for signalling, modulates signal output from the receptors and thereby serves as a potential means by which to elicit isoform-specific responses.

Histidine kinases typically participate in ‘two-component’ signalling systems. The standard two-component system, as initially discovered and characterized in prokaryotes, consists of a membrane-localized His kinase for signal perception and a response regulator that mediates signal output from the system (reviewed in Stock *et al.* 2000; Schaller and Kieber 2002). In response to an environmental stimulus, the His kinase autophosphorylates on a highly conserved His residue present in the His kinase domain of the receptor (reviewed in Mizuno 1997; Stock *et al.* 2000; Gao and Stock 2009). This phosphate is then transferred to the aspartate residue located in the receiver domain of the response regulator, which frequently functions as a transcription factor. In eukaryotes such as plants, fungi and slime moulds, a modified version of the two-component system termed a multi-step phosphorelay is most common (reviewed in Schaller *et al.* 2008, 2011). The multi-step phosphorelay often involves three proteins: (i) a hybrid His kinase containing both a His kinase and a receiver domain; (ii) a His-containing phosphotransfer (HPt) protein; and (iii) a response regulator. In the multi-step phosphorelay, the signalling phosphate is transmitted in sequence from His to Asp within the hybrid His kinase, then to the His in the HPt protein and finally to the Asp in the response regulator. Plants contain all the signalling elements necessary to propagate a hormonal signal through a multi-step phosphorelay but, to date, the primary role for this signalling system in plants appears to be in mediating responses to the phytohormone cytokinin (reviewed in Schaller *et al.* 2008).

One of the most intriguing features of the ethylene receptors found in monocots and dicots is that they exist in two subfamilies: members of subfamily 1 containing a highly conserved His kinase domain and members of subfamily 2 containing a diverged His kinase domain (reviewed in Bleecker 1999; Chang and Stadler 2001; Schaller and Kieber 2002). The ancestral form of the ethylene receptor would have contained His kinase activity and was probably acquired by plants from the endosymbiote that gave rise to the chloroplast (Rodriguez *et al.* 1999; Mount and Chang 2002). Genomic analysis of the moss *P. patens* reveals that all members of its ethylene-receptor family contain conserved His kinase domains, suggesting that the ethylene receptors of early land plants rely more upon the two-component signalling pathway than do the evolutionarily younger monocots and dicots (reviewed in Binder *et al.* 2012). In higher plants we thus appear to be witnessing a divergence toward a functionality that is independent of His kinase activity. Such a divergence is not exclusive to the ethylene receptors; the phytochromes of higher plants also originated from an ancestral His kinase but have now diverged so substantially that they completely lack His kinase activity (reviewed in Rockwell *et al.* 2006).

*In vitro* analysis of the *Arabidopsis* ethylene receptors confirms the diverged kinase activity of the subfamily 1 and 2 receptors. The subfamily 1 receptors ETR1 and ERS1 possess functional His kinase domains and autophosphorylate on the conserved His residue, indicating that they could initiate a multi-step phosphorelay in a similar fashion to the bacterial two-component systems (Gamble *et al.* 1998; Moussatche and Klee 2004). In contrast, the subfamily 2 receptors ETR2, ERS2 and EIN4 function as Ser/Thr kinases (Moussatche and Klee 2004). ERS1, when examined *in vitro*, was also found to possess Ser/Thr kinase activity, suggesting that it may now be a bi-functional kinase (Moussatche and Klee 2004). Ser/Thr kinase activity has also been detected *in vitro* for subfamily 2 receptors from tobacco and rice (Xie *et al.* 2006; Wuriyanghan *et al.* 2009; Zhang *et al.* 2010).

The fundamental differences in receptor kinase activity are suggestive that their enzymatic activity could be a significant element in controlling signal output. The major work in this area has focused on the His kinase activity found in the subfamily 1 receptors, this focus arising in part because ETR1 was the first receptor to be identified and in part because of the atypical presence of a His kinase in a eukaryote. Resolving the signalling role of His kinase activity has not been as simple to accomplish as one might at first assume. Ideally, such studies should be performed in a genetic background that lacks the endogenous His kinase activity of ETR1 and ERS1. However, although LOF mutations were isolated in *ETR1* by Hua and Meyerowitz (1998), an additional 4 years were required to identify an insertion allele in *ERS1* (Zhao *et al.* 2002; Hall and Bleecker 2003; Wang *et al.* 2003; Qu *et al.* 2007). The subfamily 1 double-mutant *etr1-7;ers1-2* was constructed and found to exhibit a constitutive ethylene-response phenotype, consistent with the role of the receptors as negative regulators of the pathway (Wang *et al.* 2003). The mutant phenotype was rescued by subfamily 1 receptors but not by subfamily 2 receptors, demonstrating a functional difference between the receptor subfamilies, one possibility being their difference in kinase activity. However, a kinase-inactive version of ETR1 also rescued the mutant phenotype, indicating that ethylene signalling could operate independently of the receptor His kinase activity (Wang *et al.* 2003).

But the role of His kinase activity in ethylene signalling was not to be resolved that simply. Subsequent analyses revealed that the *ers1-2* allele used in the study by Wang *et al*. (2003) was not a complete null (Xie *et al.* 2006; Qu *et al.* 2007). Thus the *etr1-7;ers1-2* background would still have had residual His kinase activity, preventing an accurate assessment of the degree to which His kinase activity contributes to signal output by the receptors (reviewed in Lin *et al.* 2009). The question on the role of His kinase activity was recently re-examined, employing the same basic approach used by Wang *et al*. (2003), but taking advantage of a newly isolated *ers1-3* null mutation in *ERS1* (Hall *et al.* 2012). Results from the new study confirmed one of the key findings of Wang *et al*. (2003), namely that His kinase activity is not absolutely required for signalling by the receptors, either for repressing ethylene responses in air (i.e. absence of ethylene) or for inducing ethylene responses after ethylene binds to its receptors. However, use of the new *etr1-9;ers1-3* background did reveal that, although not absolutely required, the His kinase activity of ETR1 modulates output from the receptors. Kinase-inactive lines were less responsive to ethylene based on the triple response of dark-grown seedlings as well as root growth inhibition in light-grown seedlings. Transcriptome analysis revealed broad effects on ethylene-regulated gene expression, consistent with a decrease in ethylene sensitivity of the kinase-inactive lines (Hall *et al.* 2012).

His-Asp phosphorylation activity has also been found to play a role in the ability of receptors to mediate growth recovery following exposure to ethylene (Binder *et al.* 2004; Kim *et al.* 2011). Those receptors with receiver domains (ETR1, ETR2 and EIN4) facilitate this growth recovery response, while those lacking receiver domains (ERS1 and ERS2) have no effect on the response. Based on complementation studies using mutant and chimeric receptors, normal growth recovery requires ETR1 His kinase activity and phosphotransfer through the receiver domain of ETR1, ETR2 or EIN4 (Binder *et al.* 2004; Kim *et al.* 2011). This analysis demonstrates how His kinase activity involving a subset of the receptors can play a role in mediating a specific physiological response.

Although not characterized as extensively as the His kinase activity of the receptors, there is also beginning to be evidence that Ser/Thr kinase activity in the subfamily 2 receptors could play a role in signalling. The best evidence for such a role comes from the analysis of the subfamily 2 receptor NTHK1 of tobacco (Chen *et al.* 2009). The authors of this study identified mutations of the receptor that eliminated its kinase activity and examined its functionality by overexpressing the transgene in *Arabidopsis*. Unlike wild-type NTHK1, overexpression of kinase-inactive NTHK1 failed to confer reduced ethylene sensitivity or increased salt sensitivity on *Arabidopsis*. While there are obvious caveats to this study, it does suggest that the Ser/Thr kinase activity of NTHK1 affects some level of functionality within the receptor.

Autophosphorylation of the receptors on His and Asp residues, arising from the His kinase activity of the subfamily 1 receptors, represents one obvious mechanism for altering the phosphorylation status of the receptors, but it is apparently not the only such mechanism. A recent study involving the ethylene receptors of tomato demonstrates that ethylene receptors are phosphorylated at multiple sites in a ligand-dependent manner (Kamiyoshihara *et al.* 2012). For this analysis, the Phos-tag PAGE system was employed, which separates phosphorylated from non-phosphorylated forms of a protein and also allows for resolution based on the degree of phosphorylation (Kinoshita *et al.* 2006). The subfamily 2 receptor LeETR4 and subfamily 1 receptor LeNR are both phosphorylated in the absence of ethylene, exposure to ethylene resulting in a decrease in the level of phosphorylation. Changes in the phosphorylation state correlated with changes that occur in tomato fruit during the ripening process. Significantly, LeETR4 exhibited a higher degree of phosphorylation than LeNR, pointing to isoform-specific differences in the phosphorylation status, potentially arising from differences in phosphorylation sites. The conditions employed for this analysis, as well as the fact that multiple phosphorylation states were detected for the receptors, are consistent with receptor phosphorylation occurring on Ser/Thr residues. Two potential sources, not mutually exclusive, are likely candidates for the observed receptor phosphorylation. First, phosphorylation could originate from the Ser/Thr kinase activity found in subfamily 2 receptors and thus be an example of autophosphorylation. Second, phosphorylation could originate from CTR1, which associates with both subfamily 1 and 2 receptors, the Ser/Thr kinase activity of CTR1 being suppressed by ethylene in a manner consistent with the observed changes in phosphorylation status of the tomato receptors.

These studies support the receptors having fundamental differences in the types of kinase activity they possess as well as having multiple sites for phosphorylation, these differences potentially facilitating sub-functionalization so that the receptors can participate in non-overlapping pathways for signal output. We envision two primary mechanisms by which kinase activity affects signal output from the receptors: (i) by affecting physical interactions with other proteins and (ii) by phosphorylating other proteins to regulate their activity. Evidence supports His kinase activity regulating signal output from the ethylene receptors by both these mechanisms, but the same general mechanisms may also apply to Ser/Thr kinase activity.

Phosphorylation is a common mechanism to elicit conformational changes in proteins as well as to modulate interactions between proteins. The ethylene receptors interact with each other, CTR1 and EIN2; additionally, ETR1 interacts with RTE1. Thus multiple opportunities exist for autophosphorylation of the receptors to affect the activity and/or function of associated proteins. *In vitro* analysis of the interaction between ETR1 and EIN2 supports this possibility, suggesting that His phosphorylation of ETR1 reduces its affinity for EIN2 (Bisson and Groth 2010). In addition, *Arabidopsis* lines containing His kinase-inactive ETR1 exhibit decreased sensitivity to ethylene, a phenotype that could be due to receptor autophosphorylation playing a role in regulating the activity of the associated CTR1 (Hall *et al*. 2012).

Another likely mechanism by which receptor His kinase activity could modulate downstream signalling is through a His-Asp phosphorelay involving downstream phosphotransfer proteins and response regulators. Support for such a possibility comes from evidence that the ethylene receptors of *Arabidopsis* interact with phosphotransfer proteins (Urao *et al.* 2000; Scharein *et al.* 2008) and recent *in vitro* data indicating that the affinity of the receptor ETR1 for the phosphotransfer protein AHP is phosphorylation dependent (Scharein and Groth 2011). The response regulator ARR2 has been implicated in modulating the ethylene response (Hass *et al.* 2004; Mason *et al.* 2005). The possibility of a CTR1-independent pathway, such as a two-component phosphorelay, is supported by the finding that *ctr1* mutants still exhibit a residual ethylene response (Hall and Bleecker 2003; Larsen and Cancel 2003). Functions within the multi-step phosphorelay would require the initiation of His phosphorylation from the subfamily 1 receptors, but subsequent transfer to a receiver domain, which in *Arabidopsis* involves some members of subfamily 2. In monocots, there is an even more interesting division in structure between the subfamily 1 and 2 receptors, the subfamily 1 receptors containing the conserved His kinase domains but no receiver domains, the subfamily 2 receptors containing diverged His kinase domains but also having canonical receiver domains (Yau *et al.* 2004; reviewed in Binder *et al.* 2012). In monocots, one would therefore predict that subfamily 1 receptors would autophosphorylate on the conserved His residue but this phosphate would then need to be transferred to the receiver domain of a subfamily 2 receptor before being passed on to downstream elements in the two-component signalling system. What is currently unclear is whether ethylene binding to the receptors activates or inhibits their His kinase activity, *in vitro* analysis suggesting inhibition but genetic analysis suggesting activation (Voet-van-Vormizeele and Groth 2008; Hall *et al.* 2012).

## A Current Model for Ethylene Signal Transduction

Our understanding of the mechanism for ethylene signalling through the CTR1-dependent pathway was significantly advanced in 2012. Genetic analysis had identified the ethylene receptors, CTR1, EIN2, and the EIN3 transcription factor family, double-mutant analysis then serving to order these elements within the well-known signal transduction pathway (Fig. 1). However, several key mysteries in this pathway remained unsolved. First, how is the signal from CTR1 transmitted to EIN2? The similarity of CTR1 to the Raf family of MAPKKKs suggested that a MAPK cascade might operate between CTR1 and EIN2, but although candidates for such a signalling cascade have been proposed (Novikova *et al.* 2000; Ouaked *et al.* 2003; Yoo *et al.* 2008), the results are controversial due to the subsequent discovery that the proposed MAPKK and MAPK signalling elements regulate ethylene biosynthesis (Liu and Zhang 2004; Joo *et al.* 2008; Xu *et al.* 2008; Zhao and Guo 2011). Second, how is the signal transmitted from the ER-localized EIN2 to the nuclear-localized transcription factors? Here the spatial gap in subcellular signalling also suggested that some as yet unknown factor might mediate signalling between EIN2 and the nucleus.

The solution to these mysteries appeared over the course of a couple of months in three papers (Ju *et al.* 2012; Qiao *et al.* 2012; Wen *et al.* 2012). CTR1, rather than operating through an intermediary MAPK cascade, directly phosphorylates EIN2 to inhibit its activity. The most significant sites of EIN2 phosphorylation are on Ser645 and Ser924 (Chen *et al.* 2011; Ju *et al.* 2012; Qiao *et al.* 2012), with Ser924 playing a predominant role in EIN2 regulation (Ju *et al.* 2012). Following ethylene binding to the receptors, CTR1 becomes inactivated, resulting in the dephosphorylation and proteolytic cleavage of EIN2, the cleaved C-terminal portion of EIN2 then translocating to the nucleus to regulate transcriptional events (Ju *et al.* 2012; Qiao *et al.* 2012; Wen *et al.* 2012). These discoveries offer a more streamlined vision of the CTR1-dependent signal transduction pathway and, furthermore, redirect the search for missing regulatory elements toward EIN2-targeted proteases and phosphatases.

**Figure 4.**
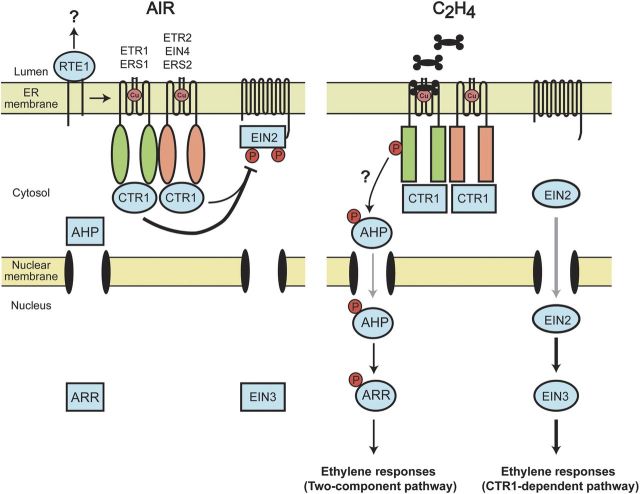
Model for ethylene signal transduction in *Arabidopsis*. The current model for signalling through the CTR1-dependent pathway is shown. In the absence of ethylene, the ethylene receptors activate CTR1, which phosphorylates and inhibits EIN2. In the presence of ethylene, the receptors inactivate CTR1, potentially through propagation of conformational changes in the receptor–CTR1 protein complex. EIN2 becomes dephosphorylated, which results in proteolytic cleavage and release of the C-terminal domain of EIN2. The C-terminal domain of EIN2 translocates to the nucleus, resulting in activation of EIN3 and the transcriptional response to ethylene. Two potential alternative pathways for ethylene signalling are also depicted (indicated by ‘?’). A two-component signalling pathway, initiated by the subfamily 1 receptors and involving phosphotransfer proteins (AHPs) and response regulators (ARRs), may mediate a subset of the ethylene responses. RTE1 may also facilitate ETR1 kinase kinase kinases signal output through a CTR1-independent pathway. Circles indicate the active forms of proteins and rectangles the inactive forms. The thickness of arrows indicates the relative contributions to the ethylene response. Grey arrows indicate translocation from the cytosol to the nucleus of the indicated proteins.

In Fig. 4, we present a current model of ethylene signal transduction incorporating these recent developments in our understanding of the CTR1-dependent pathway. The gaseous hormone ethylene is perceived in plants by a family of ethylene receptors (ETR1, ETR2, ERS1, ERS2 and EIN4 in *Arabidopsis*) predominantly localized to the ER membrane. The receptors regulate the CTR1-dependent pathway in an overlapping manner through their physical association with CTR1, stimulating the kinase activity of CTR1 in the absence of ethylene (air). Although the receptors have overlapping roles in the regulation of CTR1, the subfamily 1 receptors of *Arabidopsis* play a more predominant role than the subfamily 2 receptors in CTR1 regulation. Activation of CTR1 results in phosphorylation and inhibition of EIN2 activity, to suppress the ethylene response. Ethylene binding induces a conformational change in the receptors, resulting in the inactivation of CTR1 and the dephosphorylation of EIN2. As a result, the C-terminal portion of EIN2 is proteolytically cleaved, migrates to the nucleus, and there through an unknown mechanism activates the EIN3 family of transcription factors to initiate the transcriptional response to ethylene.

Also included in the model is a conjectured two-component signalling pathway with phosphotransfer protein and type B response regulator (Fig. 4). The two-component pathway is shown activated in the presence of ethylene, based on the finding that His kinase activity of ETR1 facilitates the ethylene growth response in *Arabidopsis*, and represents a means by which the subset of subfamily 1 receptors can potentially initiate signalling through a CTR1-independent pathway. Thinner arrows are used to indicate that the two-component pathway would play a less pronounced role in signalling than the CTR1-dependent pathway.

Several protein–protein interactions are emphasized in the model (Fig. 4). First, there is the interaction of the receptors with CTR1. Second, the receptors are shown interacting with each other, an interaction that may allow for increased sensitivity to ethylene through propagation of ethylene-induced conformational changes within a receptor cluster as well as facilitating *trans*-phosphorylation between His kinase domains and receiver domains of different receptors. Third, the interaction of RTE1 with ETR1 is indicated, an interaction that facilitates the ability of ETR1 to suppress the pathway in the absence of ethylene binding (in air) and which also serves as a prime example of how sub-functionalization can be accomplished by assembly of different receptor signalling complexes. RTE1 may also participate in a CTR1-independent signalling pathway.

## Sources of Funding

This work was supported by the Division of Chemical Sciences, Geosciences, and Biosciences, Office of Basic Energy Sciences of the US Department of Energy under grant no. DE-FG02-05ER15704 (G.E.S.) and National Science Foundation under grant no. MCB-0918430 (B.M.B.).

## Contributions by the Authors

All authors contributed to the writing of the manuscript. S.N.S. and X.W. should be considered as equal authors.

## Conflict of Interest Statement

None declared.
